# Supplementary material for: T-box3 is a ciliary protein and regulates stability of the Gli3 transcription factor to control digit number
Source: eLife. 2016 Apr 5;5:e07897. doi: 10.7554/eLife.07897 (PMC4829432; doi:10.7554/eLife.07897)
Supplement: Supplementary file 5. — DOI: http://dx.doi.org/10.7554/eLife.07897.047 [file elife-07897-supp5.docx]

**Supplementary File 5. qPCR primers**

Epha3 Fwd GTTGGTGTCACTGTGGTTGG

Epha3 Rev TTCTCACATCCCTCCAGGAC

Zic3 Fwd GGTCAACCATATCCGAGTGC

Zic3 Rev AACCGTCTGTCACAGCCTTC

Hoxd13 Fwd AGGGGTCCCATTTTTGGA

Hoxd13 Rev TGGTGTAAGGCACCCTTTTC

Histone Fwd GTGCTTAAGAGTCCACTATGAGGG

Histone Rev TCCACTCGCAATCATATACTTAGG

Actin Fwd gctcgaagtctagagcaacatagc

Actin Rev TGACAGACTACCTCATGAAGATCC

Sufu Fwd GGTTACCGCTATCGTCAAGTACTG

Sufu Rev ATGGGCACTGTCCGTAGTAGT

Kif7 Fwd GGCTTCAATGCCACCGTCTT

Kif7 Rev ATGCACCAGGCAGTCCAG

Spop Fwd CGCTTTACCTGTTGTTGGTCAGC

Spop Rev GCACGAACCTATAAGCTCGCTG

Ptch2 Fwd TGGAGCCACCTTGGTACAAGA

Ptch2 Rev TGTCACTAGAGCCACCTCGTACA

Osr1 Fwd tgtagcgtcttgtggacagc

Osr1 Rev gcgaccttacacctgtgacat

Dkk1 Fwd CTGAAGATGAGGAGTGCGGCTC

Dkk1 Rev GGCTGTGGTCAGAGGGCATG

Tbx2 Fwd tcctgctaatggacatcgtg

Tbx2 Rev agacataggtgcggaaggtg

Gli1 Fwd GGATATGATGGTTGGCAAGTGCC

Gli1 Rev TCCAGCATCCCCAACAGGTG

Cntfr Fwd GTGAATTCGTCAAAGGTGAT

Cntfr Rev CTACATCCCCAATACCTACA

Fgf8 Fwd GGCGCAGAGACAGGTCTCTAC

Fgf8 Rev CGCGCTGATGCTGGCGCGTCTT

Fgf4 Fwd TACTGCAACGTGGGCATCGGA

Fgf4 Rev CCACTCCGAAGATGCTCACCAC

Ptch1 Fwd GGCAGGAGGAGTTGATTGTGG

Ptch1 Rev CATAGTCGTAGCCCCTGAAGTG

Grem1 Fwd catacactgtgggagcgttg

Grem1 Rev gctccttgggaacctttctt

Gli3 Fwd gctcttcagcaagtggttcc

Gli3 Rev ttgctgtcggcttaggatct

Shh Fwd aaagctgacccctttagccta

Shh Rev ttcggagtttcttgtgatcttcc

Pkdcc Fwd TCTTCTTCACATACCTCCTGCCAC

Pkdcc Rev CTTGTAGAGTTCTGCAGGTACTGC
